# Evaluating the components of social and economic resilience: After two large earthquake disasters Rudbar 1990 and Bam 2003

**DOI:** 10.4102/jamba.v9i1.368

**Published:** 2017-04-25

**Authors:** Amir Bastaminia, Mohammad R. Rezaei, Mohammad H. Saraei

**Affiliations:** 1 Department of Geography, School of Humanities, Yazd University, Iran

## Abstract

Extensive damages of natural disasters have made resilience a focus of disaster management plans in order to limit damages. The aim of this study was a comparative evaluation of social and economic resilience in Bam and Rudbar. This applied research attempted to quantify and compare different dimensions of social and economic resilience in Bam and Rudbar with a descriptive-analytical method. Cochran’s formula determined the sample size as 330 households from both cities (a total of 660 households). The indicators of social and economic resilience were identified from the literature, and then data were collected through a field study using questionnaires. Data were analysed using multiple linear regression and feed-forward multilayer perceptron artificial neural network. Results denoted that several resilient-related socio-economic features were significantly different for Bam and Rudbar cities, such as the number of earthquakes experienced, length of stay in current neighbourhood and mean individual and household income. Mean social and economic resilience scores were significantly higher for Rudbar (216.3 ± 33.4 and 30.6 ± 7.3) compared to Bam (193 ± 26.5 and 29.4 ± 7.07) (*p* < 0.05). In addition, linear regression indicated that an increase in education level of the household head, length of stay in current neighbourhood and household income could result in an increase in social and economic resilience of the households under study. Neural network analysis revealed that social capital and employment recovery are the most and least effective factors, respectively, in both cities. In the population under study, social component, namely, social capital, was the most important determinant of resilience.

## Introduction

Natural disasters have always been global issues, where unawareness and lack of preparedness can lead to property damage as well as social, economic, environmental and psychological damages (O’Brien, Sygna & Haugen 2004).

Until the 1980s, theoretical literature of crisis management was focused on approaches to reduce vulnerability to and develop coping strategies for disasters. By the end of 1980s and especially in the 1990s, social scientists started criticising this approach. They believed that vulnerability has a social dimension and should not be limited to human and property damage (Blaikie et al. 2014). Since then, researchers have struggled to change the dominant paradigm of the crisis management. Therefore, nowadays disaster management attitudes have experienced a substantial change all around the world such that the prevailing view now is aimed at increasing resilience to disasters rather than merely reducing vulnerability (Gilpin & Murphy 2008).

In the new paradigm, reactivity and single agent (state-based) vision have changed into deterrence and participation (Turner 2010).

The word ‘resilience’ was first suggested in Hyogo World Conference on Disaster Risk Reduction 2005 (Birkmann et al. 2010). Gradually, this notion found its proper place in both theory and practice of reducing disaster risks and was discussed with various viewpoints such as resilient society, resilient livelihoods and resilient ecosystem (Becker 2014). Over time, in many countries, this approach entered the hierarchy of crisis management planning, in both urban and local scales (Coaffee & Lee 2016).

This reorientation was in order to better serve the interests of society with an improved and influential management of urban development (Brenner, Marcuse & Mayer 2012). So in recent years, disaster reduction institutions and agencies have further focused on achieving a resilient society; in the meantime, natural disasters and coping with earthquakes are of high priority because of extensive damage and widespread social disorders (Bristow 2010).

Thus, given the geographic location, numerous active faults of the area, numerous historical earthquakes (especially the 1990 earthquake in Bam and the 2003 earthquake in Rudbar) and other tectonic and geological evidences, there is a considerable risk of destructive earthquakes in the cities of Bam and Rudbar. Furthermore, several reasons indicate that the event of another serious earthquake would lead to irreversible loss and damage in these cities. Some of these reasons are inconsistent and improper urban growth especially during recent decades, construction on the boundary of the fault and areas prone to geological instabilities as well as river banks, lack of earthquake resistance design and constructions, lack of planning and operational risks management capabilities. As a result, it is essential to study, understand and analyse resilience, especially the social and economic dimensions of resilience, in the earthquake-stricken cities of Bam and Rudbar in Iran. Thus, the aim of this study was evaluation and comparison of the components and factors affecting social and economic resilience in earthquake-stricken cities of Bam and Rudbar.

## Theoretical foundations

### Resilience: Concept and definition

The term ‘resilience’ originates from the Latin word *resilio*, meaning ‘bounce back’ (Manyena 2014). Resilience has been linked to a number of basic sociological concepts, including crisis, economic poverty, sustainability and vulnerability (Thieken et al. 2014).

There is still debate about a main perspective for resilience; some believe it is an ecologic concept, while others attribute it to physics (Brooks 2006). Nevertheless, it is frequently stated that resilience studies are derived from laws of psychology and psychiatry in the 1940s (Waller 2001). The works of Emmy Werner, Norman Garmezy and Michael Rutter are mostly accredited for initial development of resilience (Zhou, Wan & Jia 2010).

In 1973, after a major publication by Holling, entitled ‘Resilience and Stability of Ecological Systems’, it became a widely used term (Ainuddin & Routray 2012).

Resilience is a new analytical dimension to disaster risk assessments (Bujones et al. 2013). It may be tempting to frame resilience in binary terms of presence or absence, whereas it should be considered as a process or outcome. Nevertheless, in every aspect of life, there is resilience to a certain extent (Pietrzak & Southwick 2011).

As per Kim-Cohen and Turkewitz (2012), resilience is a function of one’s development and interaction with environmental changes over time. To this end, individuals’ resilience level is dependent on resources, cultures, religions and institutions.

Carpenter et al. (2012) believe that resilience is not a state but more of a process, a process that starts with a form of disorder and continues with adaptation and development. Therefore, if the change or the disorder were to be a threat, a natural response would be an attempt to take the system back to normal, or the pre-crisis condition. On the other hand, if the same change can result in development or new facilities, it is desirable to redirect the system with innovative methods (Bastaminia et al. 2016).

Moberg and Simonsen (2011) have demonstrated the importance of learning resilience; in other words, there are lessons to be learned from each crisis and resilience is about the capacity to take advantage of these experiences in future (Moberg & Simonsen 2011). Table 1 contains some definitions of ‘resilience’ used by various researchers.

**TABLE 1:** Definitions of ‘resilience’.

Authors	Definitions
Holling, Gunderson and Peterson (2002)	The amount of disturbance that can be absorbed before the system changes its structure through modification of variables and processes that control its performance.
Pelling (2003)	The ability of the system attributes to withstand or adapt to hazard stress.
Adger et al. (2005)	The capacity of ecological systems to absorb disturbances in order to maintain necessary structures, processes and feedbacks of the system.
Cutter, Burton and Emich (2010)	The capacity to absorb basic functions and to rebound from an event.
Zolli and Healy (2012)	The methods to manage in an unbalanced world.
Boon et al. (2012b)	A dynamic process of coping and responding to adversity, while maintaining a healthy level of functioning.
Turner (2013)	The process of strengthening the capacity of population, communities, organisations and forecasting, prevention, recovery and change of cities in the face of shocks or stresses.
Birkmann et al. (2013)	Capacity of the affected community or ecosystem to absorb and restore the negative effects.

Note: Please see the full reference list of the article, Bastaminia, A., Rezaei, M.R. & Saraie, M.H., 2017, ‘Evaluating the components of social and economic resilience: After two large earthquake disasters Rudbar 1990 and Bam 2003’, *Jàmbá: Journal of Disaster Risk Studies* 9(1), a368. https://doi.org/10.4102/jamba.v9i1.368, for more information.

### Social resilience

Social resilience has multiple definitions and all of them sought to acknowledge the ability of institutions, individuals, organisations and communities to endure, absorb, cope and adjust to the environment against various social threats (Keck & Sakdapolrak 2013). Adger (2000) proposed the first definition of community resilience as the ability of a society to cope with and restore from external traumas and internal disorders of the infrastructures such as political, social, and environmental change.

Lorenz (2013) suggested a set of capacities for resilience: (1) coping capacity, (2) adaptive capacity and (3) transformation capacity. The main objective of community resilience is to improve the capacity and skills of individuals, groups and organisations in coping with disturbances (Obrist, Pfeiffer & Henley 2010). As for the personal resilience, community resilience should encompass economic, organisational, social and ecologic aspects of the society.

Social resilience is concerned with environmental stability and sustainability. Social instability could be manifested through population displacement and vice versa (Boon et al. 2012a). Buckle, Mars and Smale (2000) had studied the new approaches to social resilience and vulnerability. They had pointed out factors that will help individuals, households, groups and local communities to overcome negative consequences of disasters. These factors are known to increase social resilience of the society (Table 2).

**TABLE 2:** Components and characteristics of social resilience.

Components	Characteristics
Shared values, plans and aspirations of the local community	This includes sharing positive attitude towards the future, commitment to community as a whole and agreement of community goals; such as cultural commonalities.
Social infrastructure establishment	This includes information channels, social networks and organisations in the local community.
Positive economic and social trends	This includes growing or stable population, a healthy economic base.
Economic and social sustainability	This includes a capacity for the community to disaster management.
Cooperation	This includes cooperation among individuals, institutions and local community groups by sharing skills, experiences, knowledge resources and common goals in order to achieve innovation and increase capacity for disaster prevention and preparedness.
Interests of the local community	This is when there are socially diverse groups sharing common issues and interests, skills or expertise such as religious and cultural interests.
Network establishment	This includes agreed and stable networks among people and groups that facilitate exchange of information and sharing of resources. Thus, improving skills, time and effort to planning and preparation.
Resources and skills	Access to local resources and skills is directly associated with strategic planning. This leads to local community support in case of emergency. The support can be in terms of resources, skills and costs.

*Source*: Buckle, P., Mars, G. & Smale, S., 2000, ‘New approaches to assessing vulnerability and resilience’, *Australian Journal of Emergency Management* 15(2), 8–14

Accordingly, increasing the strength of a city is to increase the strength and content of communications between people and organisations. In fact, moving away from independence philosophy and acceptance of dependency culture is the key to coordination and development (Keck & Sakdapolrak 2013). Social resilience is not only strengthening the independence but also strengthening the social ties (Rezaei, Bastaminia & Saraie 2016).

### Economic resilience

Impact of natural disasters is not limited to human costs. Economic costs could also affect human well-being. From the viewpoint of economics, a natural disaster is a natural crisis if it brings about disruptions to the financial system, adversely affecting assets, inputs, outputs and employment (Hallegatte 2014). Disruptions to the financial system because of disaster are far beyond the usual financial consequences and are not easily resolved. These disruptions appear to have adverse longer-term economic impacts, such as lower exports and production, loss of income and livelihood, rationing in some sectors, declined employment and lower tax returns. Therefore, a reliable estimate of welfare impacts of disasters should include these economic losses (Martin 2012). Economic resilience provides a systematic approach to reduce vulnerability and economic loss and improve the critical disaster situation.

Rose and Krausmann (2013) describe economic resilience as the ability of a city to minimise potential disaster losses.

Martin and Sunley (2015) attempted to define urban economic resilience using four interpretations:

- Resilience is ‘rebounding’ from a disturbance.
- Resilience is recovering from a disturbance or ‘speed of recovery’.
- Resilience is ability to absorb the disturbance and stabilising system structure and function.
- Resilience is the capacity of the system to maintain basic performances through adaptation.

All these definitions focus on the notion of returning to normal rather than adapting to changes or transforming in response to changes (Martin & Sunley 2015).

## Methods

### Study site

Rudbar is situated in a mountainous area at 250 m a.s.l. and the bank of Sepid Rood in Gilan province, northern Iran. It is located at 36° 32’ – 37° 7’ north latitude by 49° 11’ – 50° 5’ east longitude. The climate is mild and humid and has an average annual rainfall of 650 mm. The Manjil–Rudbar earthquake occurred at 00:30 local time (21 hours Greenwich Mean Time [GMT]) on 21 June 1990 near Rudbar city and subsidiary villages in Gilan province and Tarom-e Olia region in north-western Zanjan province. It caused a wide-ranging damage in areas within a 100 km radius of the epicentre, which was at a depth of 16 km. According to records, about 35 000 were killed, 200 000 houses were destroyed and about 500 000 people were left homeless. The cost of damage to public infrastructures of Gilan and Zanjan provinces was extensive, too.

Bam is a city in Kerman province, south-eastern Iran. It is built 200 km southeast of the Kerman, near Kerman–Zahedan route, and has a population of 125 764 people (National Census of Population and Housing of Iran 2011). It is located at 29° 69’ north latitude by 58° 27’ east longitude, at an altitude of 1060 m a.s.l. The climate is hot and dry, and the annual average rainfall is 68 mm. On 26 December 2003, a massive earthquake struck Bam and subsidiary villages, killing over 26 000 residents and leaving more than 30 000 homeless (Statistical Centre of Iran 2013).

### Type of study

This is an applied research study with a descriptive- analytical-comparative method, designed to explore the concept and measures components of economic and social resilience (Bastaminia, Rezaei & Saraei 2017) in Bam and Rudbar. In line with the research question, first indicators of social and economic resilience were identified and agreed through literature review and opinions from supervisors and experts. Next, an operational definition of social and economic resilience was established upon considering local characteristics of Bam and Rudbar cities. Lastly, we designed a questionnaire to measure the identified factors at a household level.

**FIGURE 1:**
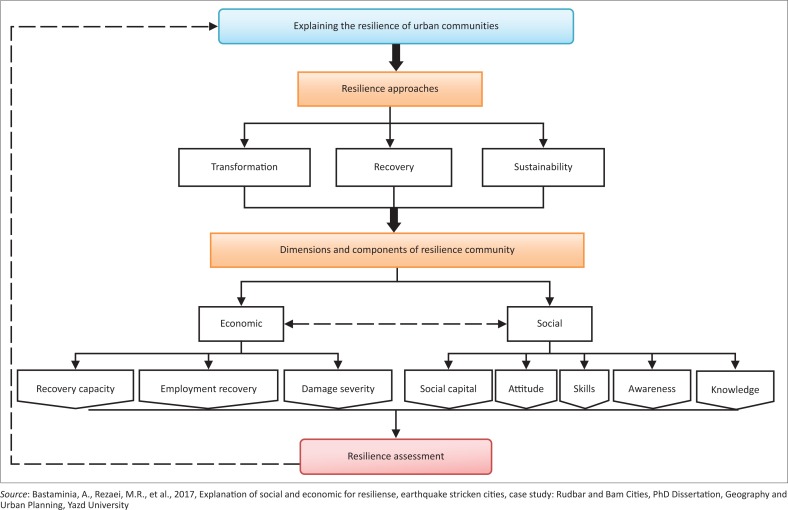
Conceptual model.

Social resilience factors include awareness, knowledge, skill, attitude and social capital. Economic resilience factors, on the other hand, include damage severity, recovery capacity and employment recovery (Figure 1). Cronbach’s alpha coefficient of the final questionnaire was 0.93, indicating high reliability.

### Study population and sampling

Target population of the current study is all households living within legal limits of Bam and Rudbar cities. Population of Bam was 125 764 consisting of 35 098 households; for Rudbar there were 3406 households amounting to a population of 10 926. The estimated sample size using Cochran’s sample size formula was 322 for Bam and 313 for Rudbar. Considering the potential sample loss, 330 samples were selected from both cities (a total of 660 households). We adopted a multistage sampling method with following steps:

- Choice of sample neighbourhoods based on clustered random sampling method: There are four regions with different socio-economic status in Rudbar and we randomly selected a neighbourhood from each region. There are six regions in Bam and one neighbourhood was randomly selected from each.
- Three hundred and thirty sample households were randomly chosen from 4 and 6 neighbourhoods proportionate to the number of households in each neighbourhood of Bam and Rudbar.
- Sample households were systematically selected from neighbourhoods.

### Data analysis

Data analysis was performed using descriptive statistics such as mean, standard deviation and frequency. Statistical inferences were made based on different tests including two-sample *t*-test, Chi-square, multiple linear regression (backward elimination) and multilayer perceptron artificial neural networks. Statistical analyses were performed using SPSS v. 22. A *p*-value of less than 0.05 was considered statistically significant.

## Results

In this section, demographic, social and economic characteristics of household heads of the two cities are compared.

According to independent sampled *t*-test results, the mean age of the household heads was significantly lower in Bam (40.50 ± 10.53), compared to Rudbar (45.80 ± 12.16) (*p* < 0.001). Economic resilience depends on several economic characteristics, which can explain the differences regarding economic resilience. Tables 3 and 4 illustrate summary statistics of these variables. Here, economic measures of the samples from two cities were compared using chi-squared and independent two-sample *t*-tests. We detected significantly different values for household and individual monthly income, approximate home value, house size (square meters), employment status and share of income spent on necessities (*p* < 0.05) (Tables 3 and 4).

**TABLE 3:** Comparison of demographic, social and economic characteristics between Bam and Rudbar cities (continuous variables).

Variable	Bam		Rudbar		*p*†
	
	Mean	s.d.	Mean	s.d.	
Age	40.50	10.53	45.80	12.16	< 0.001*
Mean individual income (monthly, $)	293.40	207.50	500.20	490.65	< 0.001*
Mean household income (monthly, $)	662.06	2104.30	4197.81	2430.95	< 0.001*
Approximate home value ($)	22701.16	15975.50	36147.34	32606.90	< 0.001*
House size (square meters)	102.3	70.20	113.86	75.08	0.037*
Length of stay in current neighbourhood (years)	24.45	13.30	34.38	11.35	< 0.001*
Number of earthquakes experienced	5.20	4.34	4.30	3.11	0.002*

† , Two-sample *t*-test.

* , *p* significant at α level less than 0.05.

**TABLE 4:** Comparison of demographic, social and economic characteristics between Bam and Rudbar cities (categorical variables).

Variable	Category	Bam		Rudbar		*P*†
	
		Frequency	Percent	Frequency	Percent	
Gender	Male	294	89.1	288	87.3	0.469
	Female	36	10.9	42	12.7	
Education	Illiterate	15	4.5	19	5.8	0.137
	Primary and secondary school	58	17.6	102	30.9	
	High school diploma	135	40.9	134	40.6	
	Associate and bachelor’s degree	112	33.9	63	19.1	
	Master’s degree and higher	10	3	12	3.6	
Employment	Employed	305	92.4	242	73.3	< 0.001*
	Unemployed	25	7.6	88	26.7	
Birth place of household head	Yes (Bam or Rudbar)	308	93.3	300	90.9	0.248
	No (other)	22	6.7	30	9.1	
Duration of stay in the neighbourhood	Less than 5 years	7	2.1	15	4.5	0.117
	5–10	8	2.4	7	2.1	
	10–15	19	5.8	21	6.4	
	15–20	48	14.5	30	9.1	
	More than 25 years	248	75.2	257	77.9	
Share of income spent on necessities	Almost all	151	45.8	49	14.8	< 0.001*
	More than half	103	31.2	139	42.1	
	Half	56	17	107	32.4	
	Less than half	15	4.5	26	7.9	
	Very little	5	1.5	9	2.7	
Physical or mental disability	Yes	22	6.7	28	8.5	0.377
	No	308	93.3	302	91.5	
Housing	Ownership	277	83.9	286	86.7	0.195
	Rental	46	13.9	38	11.5	
	Janitorial	1	0.3	4	1.2	
	Free	6	1.8	2	0.6	
Building age	Less than 10 year	260	78.8	150	45.5	< 0.001*
	10–20	50	15.2	93	28.2	
	20–30	11	3.3	52	15.8	
	More than 30 years	9	2.7	35	10.6	
Insurance	Disaster insurance	310	93.9	298	90.3	0.022*
	Medical insurance	4	1.2	6	1.8	
	None	16	4.8	26	7.9	

† , Chi-squared test.

* , *p* significant at α level less than 0.05.

The number of earthquakes experienced is another indicator of social resilience. The average number of earthquakes experienced was 5.20 (on a scale of 1–30 times) in Bam and 4.30 (on a scale of 1–10 times) in Rudbar, inferring that participants from Bam have experienced significantly more earthquakes compared to participants from Rudbar (Table 3).

Finally, only 1.2% of participants from Bam and 1.8% of participants from Rudbar had disaster insurance coverage, such as earthquake, fire and life insurance. The majority of the sample had medical insurance (Table 4). Tables 2 and 3 present a detailed summary of other variables.

### Evaluation and comparison of social and economic resilience and their components in the two cities of Bam and Rudbar

#### Social resilience and its components

Components of social resilience that associate with an increased ability to respond to and recover from disasters include awareness, knowledge, skill, attitude, and social capital. This section provides a comparison of social resilience to earthquake in Bam and Rudbar, based on these components:

- Mean score of natural disasters awareness was 43.36 and 46.06 in Bam and Rudbar, respectively. *T*-test showed that the difference is statistically significant and household heads in Rudbar are more aware of the issue, compared to those in Bam.
- Mean knowledge of participants on the resources and training about the earthquakes in Bam and Rudbar are 8.02 and 9.10, respectively. In other words, household heads in Rudbar have a significantly higher level of knowledge compared to Bam.
- Another important component of social resilience is the skill to deal with earthquakes. Mean skill scores of households in Bam and Rudbar were 22.74 and 25.47, respectively, which shows a significantly higher level of skill for families of Bam.
- Attitude and belief of individuals and families towards natural disasters are effective parameters in social resilience. In this regard, the average attitude score of the participants from Bam and Rudbar was 31.82 and 29.89, respectively. We observed a significantly higher attitude score of household heads in Bam.
- Social capital is also an important indicator of social resilience level of the communities. Mean score of social capital in Rudbar (105.77) was considerably greater than the mean score in Bam (87.62).
- Comparative analysis of social resilience data showed that social resilience of the households in Bam and Rudbar is 193.57 and 216.30 on average. In other words, households of Rudbar were more resilient compared to households of Bam. Table 5 presents more details on social resilience and its components.

**TABLE 5:** Summary statistics of the components used in measurement of economic and social resilience of the participants.

Variable	Bam				Rudbar				*t*	*p*†
	
	Mean	s.d.	Min	Max	Mean	s.d.	Min	Max		
Awareness	9.21	43.36	19	67	10.90	46.06	15	74	−3.433	0.001*
Knowledge	3.20	8.02	3	20	3.24	9.10	3	22	−4.312	< 0.001*
Skill	6.74	22.74	2	37	6.18	25.47	3	37	−5.415	< 0.001*
Attitude	5.94	31.82	17	45	6.45	29.89	15	43	4.004	< 0.001*
Social capital	16.13	87.62	52	127	20.13	105.77	60	135	−12.784	< 0.001*
Total social resilience	26.49	193.57	119	262	33.39	216.30	144	298	−9.684	< 0.001*
Damage severity	3.78	13.03	4	22	3.20	13.17	3	23	−0.621	0.535
Recovery capacity	3.37	6.83	0	15	4.12	8.35	0	15	−5.204	< 0.001*
Employment recovery	2.47	9.54	3	15	2.46	9.10	2	15	2.272	0.023*
Total economic resilience	7.07	29.37	13	49	7.29	30.63	10	49	−2.254	0.025*
Total resilience	28.82	222.95	142	297	35.95	246.94	171	329	−9.457	< 0.001*

† , Two-sample *t*-test.

* , *p* significant at α level less than 0.05.

#### Economic resilience and its components

Components of economic resilience that associate with ability of an individual or a community to recover from or adjust to the effects of a shock so that to minimise the potential losses include damage severity, recovery capacity, and employment recovery. This section provides a comparison of economic resilience to earthquakes in Bam and Rudbar based on these components:

- Damage severity is an important marker of economic resilience to assess the extent and severity of damage to properties and assets of individuals and families. The mean score of damage severity in Bam and Rudbar was 13.03 and 13.17, respectively, which shows no difference between the two cities according to the *t*-test.
- Another important component of economic resilience is the community’s recovery capacity after disaster. The mean score was 6.83 and 8.35, in Bam and Rudbar, respectively, which shows that families in Rudbar have a significantly higher score for this component.
- One more factor in economic resilience is employment recovery, the ability of families to resume financial status and return to work after disaster strike. Employment recovery score was 9.54 and 9.10, on average for families of Bam and Rudbar, respectively, indicating a greater score for Bam.

The overall economic resilience was 29.37 for Bam and 30.63 for Rudbar, specifying a higher mean score of economic resilience for families in Rudbar.

### Linear regression model

A linear regression model with backward method was employed to study the effect of demographic, social and economic variables on total resilience.

#### Regression model for Bam

The results suggested that age, birthplace and education level of the household head were associated with total resilience level. Age and education level of the household head are directly correlated with total resilience. In other words, for one unit increase in age or education level, total resilience would increase by 0.12 and 0.14, respectively. Households whose heads were born outside of Bam are 0.13 unit less resilient, compared to those born in Bam (Table 6).

**TABLE 6:** Assessing the effects of demographic, social and economic variables on total resilience of Bam and Rudbar cities.

Variables in the final model	Regression coefficients			*t*	*p*	*R*^2^

	Unstandardised	SE	Standardised			
**Bam**						
Intercept	215.25	10.63	-	20.24	< 0.001	0.04
Age	0.32	0.16	0.12	2.01	0.045*	
Birthplace of the household head	−15.12	6.35	−0.13	−2.40	0.018*	
Education level of the household head	2.62	1.10	0.14	2.40	0.017*	
**Rudbar**						
Intercept	263.50	17.21	-	15.31	< 0.001	0.48
Ethnicity	1.33	0.66	0.09	2.00	0.056	
Duration of stay in the neighbourhood	0.60	0.10	0.27	6.30	< 0.001*	
Education level of the household head	8.12	1.65	0.22	4.92	< 0.001*	
Number of employed family member (excluding household head)	3.00	1.66	0.08	1.80	0.073	
Share of income spent on necessities	3.81	1.64	0.10	2.32	0.021*	
Mental or physical disability	−17.00	5.30	−0.13	−3.22	0.001*	
Household size	−3.00	1.51	−0.09	−2.00	0.047*	
Housing status	−9.53	3.43	−0.12	−2.80	0.006*	
Building age	−12.20	1.60	−0.34	−7.64	< 0.001*	
Approximate home value	0.02	0.00	0.08	1.92	0.045*	
Number of earthquakes experienced	−1.86	1.00	−0.08	−1.93	0.034*	

Multiple linear regression (backward elimination).

* , *p* significant at α level less than 0.05.

#### Regression model for Rudbar

Regression analysis detected several factors associating with total resilience of households in Rudbar.

Duration of stay in the neighbourhood, education level of the household heads, share of income spent on necessities and approximate home value are positively associated with total resilience. Hence, for a one unit increase in any of these variables, resilience would increase 0.27, 0.22, 0.10 and 0.08 units, respectively.

In other words, the results suggest that increased education level of household heads, increased length of stay in the neighbourhood and improved economic status of households, particularly increased household income, could increase the overall resilience level of the households participating in this study.

Mental or physical disability, household size, housing status, building age and number of earthquakes experienced are negatively correlated with total resilience. More specifically, for a one unit increase in household size and number of earthquakes experienced, total resilience score reduces by 0.09 and 0.08. Interestingly, individuals with mental or physical disability are more resilient to hazard. Regarding housing status, the estimated coefficient is 0.12, meaning that compared to home ownership, individuals with rental or janitorial housing are 0.12 unit less resilient. Lastly, for a unit increase in building age, resilience decreases to 0.34 unit. Ergo, families living in a newly built house are 0.34 unit more resilient than those living in a 5–10-year-old house, and families living in a 5–10-year-old house are 0.34 unit more resilient than those living in a 10–15-year-old house, and so on (Table 6).

It is worth mentioning that the coefficient of multiple determination or *R*-squared was 0.04 and 0.48 for Bam and Rudbar regression models. Consequently, 4% of variance in total resilience of Bam and 48% of variance in total resilience of Rudbar are explained by the variance in demographic, social and economic variables.

### Neural network sensitivity analysis and prediction

Neural networks are one of artificial intelligence techniques used widely for solving different problems, including forecasting, detection and control.

In general, an artificial neural network has three types of layers: input, hidden and output. In each layer, there are a number of neurons responsible for data processing. Data enter the network in first layer, they are processed through hidden layers and the result is produced by the neurons from the output layer. Input layer works like a bridge and transfers the input data or independent variables into the network. Computations happen in the intermediate layers and output is prepared by a mathematical processor which is the activating or stimulant function. The major difference between an artificial network and other neural network models is the ability to learn. By providing independent variables as the input and the dependent variable as the target variable, the network learns to produce outputs that are very close to the actual value (target variable). This is done by constant modification of the weights and bias. In principle, the advantage of neural networks is the use of non-linear functions in the hidden layer that provides the ability to analyse any type of data. Thus, they are very useful to discover complex relationships between variables.

Among various neural network models, the multilayer perceptron neural network (MLP) is one of the most flexible and popular methods because of features such as the use of non-linear stimulant functions, multi-layered structure and choice of active learning algorithms.

In this study, we use a neural network model to describe the relationship between components of economic and community resilience and total resilience in Bam and Rudbar. Furthermore, a neural network sensitivity analysis is applied to determine the impact of input variables on output variables. Sensitivity analysis is a technique used to determine a causal model of inputs and outputs from a neural network model. In other words, sensitivity analysis orders the variables based on relative importance of the input to estimate output. Therefore, a relative importance of 0 indicates that the independent variable does not contribute to the estimation of the dependent variable, whereas a relative importance of 1 indicates that the independent variable greatly contributes to the estimation of the dependent variable.

Number of hidden layers and neurons verify the structure of the network. The number of hidden layers is selected based on the number of independent and dependent variables and factor levels. To choose the optimum number for the neurons in the input and hidden layer, one should train the network with a different number of neurons and use the trial with minimum averaged test error.

#### Type of artificial neural networks

In this study, a feed-forward multilayer perceptron network with one input layer, eight neurons (following the number of independent variables) and one output layer (following the number of dependent variables) was utilised.

#### Artificial neural network processing paradigms

Neural network architecture is as follows:

- **Rescaling:** A standard processing approach is to scale the inputs and output variables to facilitate the learning process. We standardised the data by subtracting the mean and dividing by the standard deviation to have mean 0 and a variance of 1.
- **Random number generation:** Random numbers are used for a variety of purposes in neuronal networks. To perform supervised learning one needs two types of data sets: a training set and a validation set. Here, we used 70% of the sample (231) to train and 30% (99) to test the network for each city.
- **Choice of hidden layer activation functions:** The activation function of a layer defines the output of that layer, given an input. We selected the popular hyperbolic tangent function, which converts the input data to values within the range of +1 to -1.
- **Training method:** A neural network is trained with a specific portion of data, which means calibrating all synaptic weights and building a prognostic model. The next step is to validate the network using the other portion of the sample data. In this article, we have used the batch learning, which would update the synaptic weights only after passing all training data. Hence, it generates the best predictor by learning on the full data matrix.
- **Optimisation algorithm:** This is an analytical method to estimate synaptic weights. There were two available options, scaled conjugate gradient and gradient descent. In our model, we adopted the scaled conjugate gradient that provides faster training and is only available to batch training.
- **Choice of output layer activation functions:** Considering the continuous nature of output variable (total resilience), identity activation function was preferred as our output layer represents a real-valued target.

**FIGURE 2:**
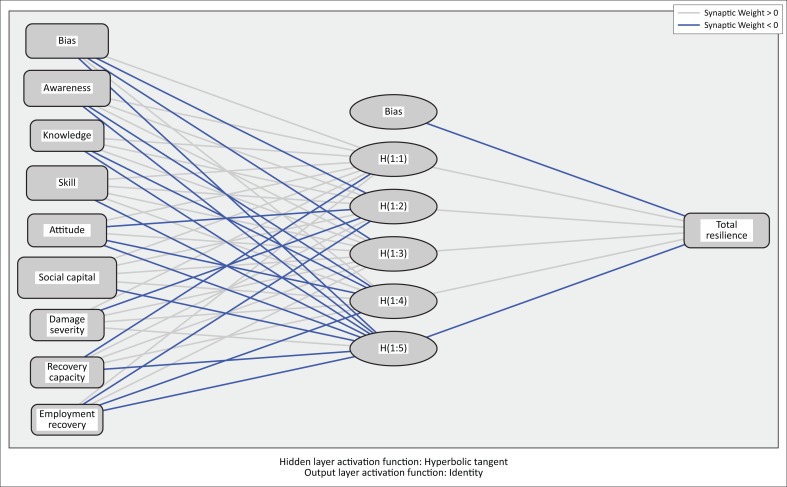
Structure of the artificial neural network of Bam including estimated synaptic weights.

**FIGURE 3:**
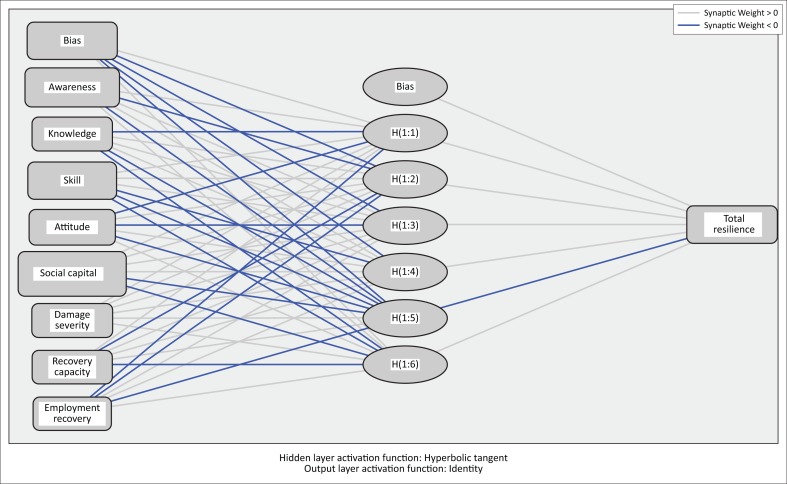
Structure of the artificial neural network of Rudbar including estimated synaptic weights.

The predictive accuracy of the models was evaluated using mean squared error (MSE) and root mean squared error (RMSE) loss functions. For Bam, a network with 8 input layers and 5 hidden layers (8, 5, 1) *and* for Rudbar a network with 8 input layers and 6 hidden layers (8, 6, 1) *performed* better. The resulting networks along with corresponding synaptic weights are illustrated in Figures 2 and 3.

Starting on the left, input layers, illustrating the independent variables, are connected to hidden-layer neurons through estimated weights. Lines that are leaving the hidden layers are connecting these neurons to the output layer, which has only one neuron because of the nature of our response variable. Dark lines indicate a negative relation, while lighter lines indicate a positive relation. Each weight symbolises the importance of the relative independent variable.

#### Neural network model results

Tables 7 and 8 depict the weights assigned to each layer through learning process. These are the output values of the network.

**TABLE 7:** Estimated coefficients by the neural network for hidden layer, Bam.

Input layer	Hidden layer				

	Neuron 1	Neuron 2	Neuron 3	Neuron 4	Neuron 5
Bias	−0.756	−0.715	−0.521	1.154	−1.304
Awareness	−0.184	0.403	0.548	−0.239	0.427
Knowledge	−0.489	0.276	−0.009	0.063	0.107
Skill	0.394	−0.090	0.420	−0.561	0.332
Attitude	−0.554	0.374	0.312	−0.060	0.151
Social capital	0.017	0.801	0.392	−0.718	0.558
Damage severity	−0.258	−0.194	0.080	−0.063	0.467
Recovery capacity	0.287	0.286	0.280	−0.354	−0.185
Employment recovery	−0.519	−0.173	0.139	0.217	0.407

**TABLE 8:** Estimated coefficients by the neural network for hidden layer, Rudbar.

Input layer	Hidden layer					

	Neuron 1	Neuron 2	Neuron 3	Neuron 4	Neuron 5	Neuron 6
Bias	−0.962	−1.188	−1.256	1.132	−0.402	−1.379
Awareness	−0.223	0.586	0.504	−0.463	0.094	0.507
Knowledge	−0.554	0.184	−0.024	−0.180	−0.109	0.228
Skill	0.154	−0.024	0.384	−0.747	0.407	0.304
Attitude	−0.525	0.254	0.304	−0.345	−0.368	−0.087
Social capital	0.103	0.862	0.449	−1.219	−0.017	0.583
Damage severity	−0.496	−0.325	0.310	−0.050	−0.166	0.175
Recovery capacity	0.354	0.356	−0.123	−0.474	−0.290	0.201
Employment recovery	−0.596	−0.424	0.258	0.080	−0.437	0.218

For Bam, as per Table 7, variables such as awareness, knowledge, skill, attitude, social capital, damage severity and employment recovery are all positively associated with total resilience. In contrast, recovery capacity negatively influences the total resilience.

As evident from Table 8, for Rudbar awareness, knowledge, skill, social capital, damage severity and employment recovery are all positively associated with total resilience. Conversely, attitude negatively contributes to total resilience score.

#### The order of importance

**FIGURE 4:**
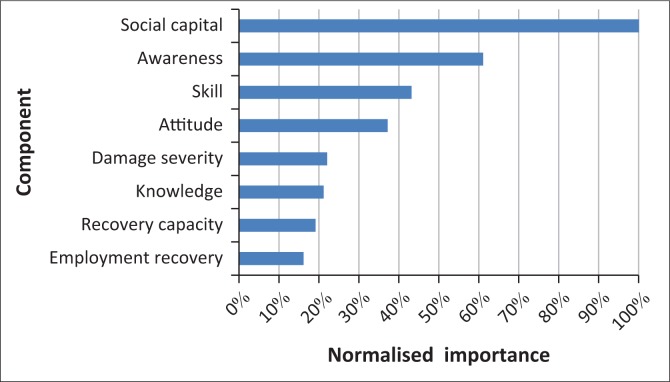
The importance of each independent variable in the neural network model.

Figure 4 compares the importance of the predictors in determining the model. Clearly, for both cities, social capital is the most important determinant. Awareness and skill are the next two important variables. Furthermore, employment recovery is the least effective index for total resilience.

## Ethical considerations

Ethical issues (including plagiarism, informed consent, research misconduct, data fabrication and/or falsification, double publication and/or submission, redundancy, etc.) have been completely observed by the authors. The Ethics Committee of Yazd University approved the study protocol. Informed consent (oral and written) of all participants was obtained.

## Discussion and conclusion

While urban resilience satisfies only if all resilience components are in a suitable or improving state, an unbalanced improvement of random components will not result in urban or even individual resilience. One important indicator of urban resilience is socio-economic progression, which is highly dependent on citizens. In this respect, the main objective of the current study was to evaluate and compare components of social and economic resilience in Bam and Rudbar cities.

According to the results, some social factors were significantly different between Bam and Rudbar, namely, the number of earthquake experiences and duration of stay in the current neighbourhood. There was no considerable difference regarding other social factors such as age and education.

As for resiliency-related economic factors, we observed that average monthly income, approximate home value and home size were higher in Rudbar families. It seems that the household economic state in Rudbar is more desirable compared to Bam.

Rudbar families had a higher score for the two dependent factors, social and economic resilience.

The findings revealed that for Bam only age and education level of the household head were directly associated with total resilience. However, several influential factors were detected in Rudbar, of which, duration of stay in the neighbourhood, education level of the household head, share of income spent on necessities and approximate home value were positively correlated with total resilience.

Neural network analysis confirmed that almost all the potential factors that were identified at the first stage of the study are related to total resilience (except for ‘recovery capacity’ in the Bam model and ‘attitude’ in the Rudbar model). In addition, social capital was introduced as the most effective indicator of resilience.

The results of neural network analysis suggest that social component of resilience, specifically social capital, was the most important indicator of resilience. This means that although elements such as the strength of buildings, economic status, earthquake-training and advertisement of the government are apparently effective in resilience, variables such as earthquake awareness, preparedness, skills, knowledge and social capital are more critical and play a key role in resilience level of households in Bam and Rudbar. Therefore, these are the main factors which should be taken into consideration to improve the resilience level in both cities.

Our results are consistent with that of Jigyasu (2004) on the role of poor skills, abilities, training, knowledge, awareness and socio-economic imbalances in increased vulnerability and resilience of rural communities in South Asia; with that of Mayunga (2007) on the development of social capital, human capital and economic capital and their effect on community resilience; with that of Ainuddin and Routray (2012) on socio-economic components leading to increased social vulnerability and decreased resilience; with that of Aldrich and Meyer (2015) on the direct effect of social factors such as improved social networking and social capital along with increased community involvement on resilience against natural disasters in coastal areas; with that of Cutter, Ash and Emrich (2014) on the assessment of resilience from social, economic and housing views in the United States; with that of Rezaie, Rafieian and Askari (2010) on social and economic components of resilience in Tehran; with that of Behtash et al. (2013) on unfavourable resilience level in Tabriz with sociocultural component as the most effective measure of resilience; and with that of Ramezanzadeh and Badri (2014) on the effect of social, cultural, economic factors on the overall resilience in Tonekabon Kile spring and Sarabrood in Kelardasht.

Based on the current knowledge, we propose the following approaches to improve resilience in Rudbar and especially in Bam:

- creating the necessary infrastructures for training courses in order to improve awareness, knowledge, attitude and skills so as to expand safety culture and improve disaster, especially earthquake, preparedness
- increasing social capital level through creation of local groups and networks, solidarity, collective action and cooperation
- expanding and reinforcing thorough scientific studies of the concept to identify disaster hazards, and risk of earthquake as a priority, and supporting science and research centres
- creating an organisation under the President’s direction to prepare and effectively confront to control crisis damage until the end of a crisis period
- pre-crisis planning and defining roles and responsibilities according to national disaster management plans
- improving awareness and prepare different social groups to reduce earthquake hazard. Conducting manoeuvres against earthquake is an essential step to identify and take measures before crisis strike, and can result in reduced damage and loss
- encouraging participation in disaster preparedness activities to get secured against and prepared for natural disasters.
